# High Prevalence of Antibodies against Canine Parvovirus and Canine Distemper Virus among Coyotes and Foxes from Pennsylvania: Implications for the Intersection of Companion Animals and Wildlife

**DOI:** 10.1128/spectrum.02532-21

**Published:** 2022-01-26

**Authors:** Caellaigh N. Kimpston, Amanda L. Hatke, Benjamin Castelli, Nathan Otto, Hannah S. Tiffin, Erika T. Machtinger, Justin D. Brown, Kyle R. Van Why, Richard T. Marconi

**Affiliations:** a Department of Microbiology and Immunology, Virginia Commonwealth University Medical Center, Richmond, Virginia, United States; b Department of Entomology, Pennsylvania State University, University Park, Pennsylvania, United States; c Department of Veterinary and Biomedical Sciences, Pennsylvania State University, University Park, Pennsylvania, United States; d USDA-APHIS Wildlife Services, Harrisburg, Pennsylvania, United States; Changchun Veterinary Research Institute

**Keywords:** *Canine distemper virus*, *Canine parvovirus*, coyote, gray fox, Pennsylvania, red fox, wildlife

## Abstract

*Canine distemper virus* (CDV) and *Canine parvovirus* (CPV) can cause deadly infections in wildlife and companion animals. In this report, we screened serum from free-ranging eastern coyotes (Canis latrans; *N *= 268), red foxes (Vulpes vulpes; *N *= 63), and gray foxes (Urocyon cinereoargenteus; *N *= 16) from Pennsylvania, USA, for antibodies (Abs) to CDV and CPV. This comprehensive screening was achieved using a commercially available enzyme-linked immunosorbent assay (ELISA)-based colorimetric assay. Abs to CDV and CPV were detected in 25.4% and 45.5% of coyotes, 36.5% and 52.4% of red foxes, and 12.5% and 68.8% of gray foxes, respectively. Abs to both viruses were detected in 9.7% of coyotes, 19.1% of red foxes, and 12.5% of gray foxes. This study demonstrates significant wildlife exposure in a northeastern state to CDV and CPV. As wildlife species continue to urbanize, the probability of spillover between domestic animals and wildlife will increase. Ongoing surveillance of wildlife for CDV and CPV exposure is warranted.

**IMPORTANCE** *Canine distemper virus* (CDV) and *Canine parvovirus* (CPV) are significant health threats to domestic dogs (Canis familiaris) and wildlife. CDV and CPV have been identified in diverse vertebrates, including endangered wildlife species. Susceptibility to these viral pathogens varies significantly among geographic regions and between host species. High morbidity and mortality have been reported with infection by either virus in susceptible species, including dogs. As humans and companion animals encroach on wildlife habitat, and as wildlife becomes increasingly urbanized, the potential for transmission between species increases. This study assessed CPV and CDV Ab prevalence in wild canids (eastern coyotes, red foxes, and gray foxes) harvested in Pennsylvania between 2015 and 2020. High Ab prevalence was demonstrated for both viruses in each species. Ongoing monitoring of CPV and CDV in wildlife and increased efforts to vaccinate dogs and prevent spillover events are essential.

## OBSERVATION

*Canine parvovirus* (CPV) and *Canine distemper virus* (CDV) are significant health concerns in domestic dogs (Canis familiaris) and diverse vertebrates, including several endangered species (1). CPV is a nonenveloped single-stranded DNA virus of the CPV-2 *Parvoviridae* family (genus *Protoparvovirus*) (2, 3). Three major lineages of CPV circulate worldwide, with CPV-2c being the dominant variant (4). CDV is a nonsegmented single-stranded RNA virus of the family *Paramyxoviridae* (genus *Morbillivirus*) (5). Phylogenetic analyses have identified 17 distinct CDV lineages (6).

CDV and CPV are highly infectious and have a broad host range. Susceptibility to overt disease varies between species. Some species develop asymptomatic infections, while others have high morbidity and mortality (7). CPV is spread through direct contact with infected animals, feces, or contaminated surfaces. CDV is primarily spread through aerosol droplets. Dogs and wild canids are particularly susceptible to infection with these pathogens due in part to their behavior, sociality, and scent communication (8). While vaccination for CDV and CPV is safe and highly effective, fatal CDV infections have been reported in vaccinated dogs infected with CDV lineages not represented in current vaccines (9). The emergence of new CDV lineages coupled with vaccine noncompliance has resulted in well-documented outbreaks and spillover events between dogs and wildlife (10–14). Interspecies transmission is a risk factor at all gradients of human habitation (urban, suburban, and rural) due to wild canids’ use of these habitats. Serological surveys of wild canids from defined geographic regions for antibodies (Abs) to CDV and CPV have been of limited sample size. To our knowledge, no recent comprehensive serological analysis of wild canids in the northeastern United States has been conducted. Understanding the exposure of wildlife to CPV and CDV is essential for devising strategies to control the interspecies spread of these deadly viruses.

**FIG 1:**
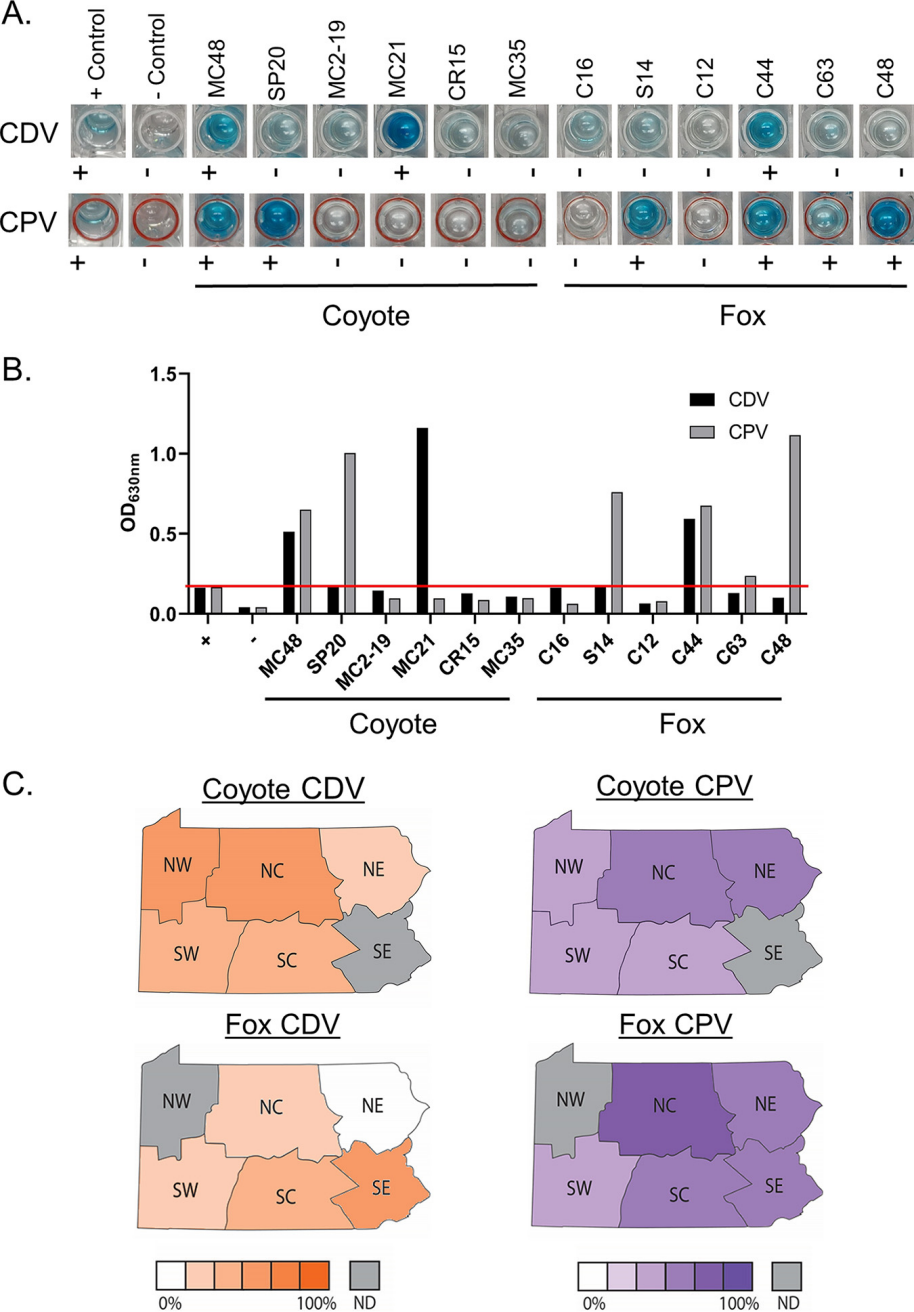
Detection of CDV and CPV Abs in serum from eastern coyotes and red and gray foxes. (A) Serum samples were screened for CDV- and CPV-specific Abs as detailed in the text. Pictures of representative ELISA plate wells are shown with animal identification numbers above each image. Visual scoring results (+ or −) are provided below each image. (B) To validate the visual scoring, the absorbance values for representative samples were determined. The positive threshold (red line) was determined based on the absorbance value of the TiterCHEK kit (Zoetis) positive control. (C) Regional differences in seroprevalence. The map divides the Commonwealth of Pennsylvania into six sectors (NW, northwest; NC, north central; NE, northeast; SW, southwest; SC, south central; SE, southeast). Each sector is color coded as indicated in the figure to reflect the number of Ab-positive animals for CDV or CPV. Gray coloration indicates insufficient data to determine percentages. Data for red and gray foxes were combined. ND, not determined.

In this study, we assessed CPV and CDV Ab prevalence in eastern coyotes (Canis latrans; *N* = 268), red foxes (Vulpes vulpes; *N* = 63), and gray foxes (Urocyon cinereoargenteus; *N* = 16) harvested in the Commonwealth of Pennsylvania. The samples were collected by the U.S. Department of Agriculture (USDA), Pennsylvania Game Commission, and Pennsylvania State University personnel in the course of wildlife damage and disease management activities and during Pennsylvania hunting and trapping seasons (years 2015, 2017, 2019, and 2020) (15). The serological analyses conducted as part of this study were performed under USDA Special Use Scientific Study Permit 48548 and a Pennsylvania State University Institutional Animal Care and Use Committee (IACUC)-approved protocol (201900871). The collection sites, collection year, sex, species, developmental stage (juvenile, 1 yr; subadult, 1 to 2 yr; and adult, >3 yr) as determined by dentition (16) and serologic status (scored as + or – for Abs to each virus) of each sample assessed are provided in Table S1. Serum samples were screened using a commercially available colorimetric-based enzyme-linked immunosorbent assay (ELISA) kit (TiterCHEK CDV-CPV; Zoetis) per the manufacturer’s protocol. Photographic images of representative ELISA plate wells are shown in Fig. 1A, with the complete set of images provided in Fig. S1. In addition to visual scoring, the absorbance (*A*_630_) values of representative samples were measured (Biotek ELx808 plate reader) and compared with the kit control values (Fig. 1B). Abs to CDV and CPV were detected in 25.4% and 45.5% of coyotes (*N* = 268), 36.5% and 52.4% (*N* = 63) of red foxes, and 12.5% and 68.8% (*N* = 16) of gray foxes, respectively (Table 1). Red foxes had a higher seroprevalence for CDV (36.5%; 95% confidence interval [CI], 25.7% to 48.9%; *N* = 63) than gray foxes (12.5%; 95% CI, 2.24% to 37.3%; *N* = 16) (*P* = 0.0652). Abs to both viruses were detected in 9.7% (95% CI, 6.7% to 13.9%) of coyotes, 19.1% (95% CI, 11.1% to 30.6%; *P* = 0.9801) of red foxes, and 12.5% (95% CI, 2.2% to 37.3%) of gray foxes. Chi-square categorical analyses were used to test for independence, and contingency tables were created and analyzed using GraphPad Prism (significance set at *P* < 0.05).

**TABLE 1:** Summary of CDV and CPV antibody screening data

Parameter	Data for:^*a*^								
	Coyote		Red fox			Gray fox			
	CDV	CPV		CDV	CPV		CDV	CPV	
Percent positive	25.4 (68/268)	45.5 (122/268)		36.5 (23/63)	52.4 (33/63)		12.5 (2/16)	68.8 (11/16)	
Positive for both	9.7 (26/268)			19.1 (12/63)			12.5 (2/16)		
Sex									
Female	19.8 (22/111)	42.3 (47/111)		30.4 (7/23)	52.2 (12/23)		0.0 (0/5)	60.0 (3/5)	
Male	19.7 (44/148)	48.6 (72/148)		40.0 (14/35)	45.7 (16/35)		10.0 (1/10)	70.0 (7/10)	
Not reported	22.2 (2/9)	33.3 (3/9)		40.0 (2/5)	100 (5/5)		50.0 (1/2)	50.0 (1/2)	
Age									
Adult	23.2 (26/112)	51.8 (58/112)		22.7 (5/22)	36.4 (8/22)		0.0 (0/14)	64.3 (9/14)	
Subadult	26.7 (23/86)	46.5 (40/86)		46.2 (6/13)	46.2 (6/13)		ND	ND	
Juvenile	27.9 (17/61)	36.1 (22/61)		10.0 (1/10)	40.0 (4/10)		ND	ND	
Not reported	22.2 (2/9)	22.2 (2/9)		61.1 (11/18)	83.3 (15/18)		66.7 (2/3)	66.7 (2/3)	
State sector									
Northwest	35.3 (18/51)	35.3 (18/51)		ND	ND		ND	ND	
North central	33.9 (21/62)	50.0 (31/62)		0.0 (0/3)	100 (3/3)		33.3 (1/3)	66.7 (2/3)	
Northeast	14.9 (14/94)	54.3 (51/94)		0.0 (0/3)	33.3 (1/3)		NC (0/1)^*b*^	NC (1/1)^*b*^	
Southwest	27.9 (12/43)	39.5 (17/43)		33.3 (1/3)	33.3 (1/3)		0.0 (0/3)	33.3 (1/3)	
South central	22.2 (2/9)	33.3 (3/9)		37.0 (17/46)	47.8 (22/46)		12.5 (1/8)	87.5 (7/8)	
Southeast	NC (0/1)^*b*^	NC (0/1)^*b*^		62.5 (5/8)	75.0 (6/8)		NC (0/1)^*b*^	NC (0/1)^*b*^	
Not reported	12.5 (1/8)	25.0 (2/8)		ND	ND		NC (0/1)^*b*^	NC (0/1)^*b*^	
Collection yr									
2015	39.3 (24/61)	47.5 (29/61)		ND	ND		ND	ND	
2017	16.7 (11/66)	42.4 (28/66)							
2020	23.5 (33/140)	46.4 (65/140)							
Not reported	NC (0/1)^*b*^	NC (0/1)^*b*^							

a Percentage of sera determined to be Ab positive (number of positive samples/total number tested).

b Due to small sample size, the percentages were not calculated. CDV, *Canine distemper virus*; CPV, *Canine parvovirus*; NC, not calculated; ND, not determined.

The large number of coyote serum samples tested in this study (*N* = 268) allowed for an assessment of Ab prevalence over time and the influence of developmental stage and sex as biological variables. The prevalence of CDV Abs was highest for serum from coyotes collected in 2015 (39.3%; 95% CI, 28.1% to 51.9%; *N* = 61), compared with 2017 (16.7%; 95% CI, 9.4% to 27.6%; *N* = 66; *P* = 0.0043) and 2020 (23.5%; 95% CI, 17.3% to 31.3%; *N* = 140; *P* = 0.0226). The CDV Ab prevalence was not influenced by developmental stage or sex (Table 1). The CPV Ab prevalence was similar throughout the collection time frame (47.5%, 42.4%, and 46.4% in 2015, 2017, and 2020, respectively). In contrast to CDV, the CPV Ab prevalence was 36% for juvenile coyotes and 51.8% for adults. When the results from different regions of Pennsylvania were compared, CDV seropositivity was highest in coyotes from the northwest (35.3%; 95% CI, 23.6% to 49.1%; *N* = 51) and north central sectors of Pennsylvania (33.9%; 95% CI, 23.3% to 46.3%; *N* = 62) (Table 1; Fig. 1). Similar to CDV, the highest CPV Ab prevalence was noted in coyotes harvested in the northeast (54.3%; 95% CI, 44.2% to 64.0%; *N* = 94) and north central sectors of the state (50.0%; 95% CI, 37.9% to 62.1%; *N* = 62) (Table 1; Fig. 1).

Fewer fox serum samples were available, but the total number was sufficient for assessing general trends. The CDV Ab prevalence was 36.5% for red foxes and 12.5% for gray foxes. The lower CDV seropositivity in gray foxes is consistent with a study of CDV Ab prevalence in foxes from Wisconsin that reported CDV Ab prevalence values of 11% and 0% for red (*N* = 57) and gray (*N* = 32) foxes, respectively (17). Gray foxes have been demonstrated to be susceptible to infection with high lethality (18). Hence, the lower number of CDV-positive gray foxes may be due to fewer animals surviving infection. In contrast to CDV, Ab prevalence for CPV was high in gray foxes (68.8%), particularly in the north central and south central sectors of the state (66.7% and 87.5%, respectively).

A subset of the coyote serum samples tested in this report was previously screened for Abs to Borreliella burgdorferi and Anaplasma phagocytophilum, the causative agents of Lyme disease and anaplasmosis, respectively (15). Of the coyote serum samples assessed in this report that were included in the study by Izac et al., 72.5% (95% CI, 67.7% to 77.5%; *N* = 265) were Ab positive for B. burgdorferi and 81.9% (95% CI, 76.8% to 86.1%; *N* = 265) were positive for Ab to A. phagocytophilum. Of the coyote samples assessed in both reports, 19.6% (95% CI, 15.27% to 24.8%; *N* = 265) and 20.0% (95% CI, 15.6% to 25.3%; *N* = 265) were Ab positive for CDV and B. burgdorferi or A. phagocytophilum, respectively. Additionally, 34.3% (95% CI, 28.9% to 40.3%; *N* = 265) and 41.1% (95% CI, 35.4% to 47.1%; *N* = 265) of the serum samples were Ab positive for both CPV and B. burgdorferi or A. phagocytophilum, respectively. To our knowledge, the sample set analyzed in this study, and the Izac study, is the first comprehensive wild canid serum panel from a northeastern state to be tested for Abs to CDV, CPV, and Ixodes scapularis-vectored pathogens.

In summary, while the interspecies transfer of biological pathogens, including CDV and CPV, is well documented, this study is the most comprehensive analysis of wild canids from an individual northeastern state. Future analyses that seek to determine the genotype of the infecting viral strains would yield valuable information relative to the potential effectiveness of current vaccines when an interspecies transfer occurs. Additional comprehensive analyses of the CDV and CPV Ab prevalence in wildlife from different geographic regions would elevate our understanding of the distribution of these viral pathogens in nature and their potential interspecies transfer between wildlife and companion animals.
