# Supplementary material for: High Prevalence of Antibodies against Canine Parvovirus and Canine Distemper Virus among Coyotes and Foxes from Pennsylvania: Implications for the Intersection of Companion Animals and Wildlife
Source: Microbiol Spectr. 2022 Jan 26;10(1):e02532-21. doi: 10.1128/spectrum.02532-21 (PMC8791182; doi:10.1128/spectrum.02532-21)
Supplement: SUPPLEMENTAL FILE 1 — Supplemental material. Download SPECTRUM02532-21_Supp_1_seq5.pdf, PDF file, 1.4 MB [file spectrum02532-21_supp_1_seq5.pdf]

Supplementary Table S1. Coyote and fox sample information and serological status

Serum ID	Sex	PA County	Collection Date	Stage of dev.	Species	CDV Ab	CPV Ab
CR-04	F	Clearfield	2/8/2015	J	Coyote	+	-
CR-06	M	Tioga	2/8/2015	A	Coyote	-	-
CR-07	F	Columbia	2/8/2015	SA	Coyote	-	-
CR-09	F	Cambria	2/8/2015	SA	Coyote	-	+
CR-10	M	Cambria	2/8/2015	A	Coyote	+	+
CR-11	M	Jefferson	2/8/2015	A	Coyote	+	-
CR-12	M	Somerset	2/8/2015	A	Coyote	+	+
CR-13	F	Washington	2/8/2015	A	Coyote	-	+
CR-14	F	Washington	2/8/2015	SA	Coyote	-	-
CR-15	F	Schuylkill	2/8/2015	A	Coyote	-	-
CR-16	M	Luzerne	2/8/2015	SA	Coyote	-	-
CR-17	M	Luzerne	2/8/2015	SA	Coyote	-	-
CR-18	M	Cambria	2/8/2015	SA	Coyote	+	+
MC012	M	Clarion	2/22/2015	J	Coyote	+	+
MC014	M	Clarion	2/22/2015	SA	Coyote	+	+
MC015	M	Clearfield	2/22/2015	A	Coyote	-	+
MC016	M	Clearfield	2/22/2015	A	Coyote	+	+
MC017	F	Washington	2/22/2015	J	Coyote	-	-
MC018	M	Erie	2/22/2015	SA	Coyote	-	+
MC019	M	Erie	2/22/2015	SA	Coyote	+	-
MC021	M	Cumberland	2/22/2015	A	Coyote	+	-
MC100	M	Mercer	2/22/2015	A	Coyote	+	-
MC113	F	Butler	2/22/2015	SA	Coyote	+	+
MC114	M	Butler	2/22/2015	A	Coyote	-	-
MC115	F	Tioga	2/22/2015	A	Coyote	+	-
MC130	NR	NR	2/22/2015	NR	Coyote	+	-

MC131	NR	Erie	2/22/2015	A	Coyote	+	+
MC148	F	Clarion	2/22/2015	J	Coyote	-	-
MC2-001	M	Clinton	2/17/2017	SA	Coyote	-	-
MC2-002	M	Clearfield	2/17/2017	A	Coyote	-	+
MC2-003	F	Susquehanna	2/17/2017	A	Coyote	-	-
MC2-004	F	Indiana	2/17/2017	SA	Coyote	-	-
MC2-005	M	Susquehanna	2/17/2017	A	Coyote	-	+
MC2-006	M	Centre	2/17/2017	J	Coyote	-	-
MC2-008	F	Northumberland	2/17/2017	A	Coyote	-	+
MC2-010	F	Crawford	2/17/2017	A	Coyote	-	-
MC2-011	F	Crawford	2/17/2017	A	Coyote	+	-
MC2-013	M	Crawford	2/17/2017	J	Coyote	+	-
MC2-016	M	Crawford	2/17/2017	A	Coyote	-	-
MC2-018	F	Elk	2/17/2017	A	Coyote	-	-
MC2-019	M	Clearfield	2/17/2017	A	Coyote	-	-
MC2-100	F	Monroe	2/19/2017	A	Coyote	-	+
MC2-101	NR	NR	2/19/2017	NR	Coyote	-	-
MC2-104	NR	NR	2/19/2017	NR	Coyote	-	+
MC2-111	F	Crawford	2/19/2017	J	Coyote	+	-
MC2-113	M	Crawford	2/19/2017	A	Coyote	-	-
MC2-119	F	Erie	2/19/2017	A	Coyote	-	+
MC2-121	M	Clinton	2/19/2017	A	Coyote	+	+
MC2-124	M	Luzerne	2/19/2017	SA	Coyote	-	-
MC2-125	F	Luzerne	2/19/2017	SA	Coyote	-	+
MC2-131	F	Susquehanna	2/19/2017	SA	Coyote	-	-
MC2-134	F	Westmoreland	2/19/2017	J	Coyote	-	+
MC2-141	M	Potter	2/19/2017	A	Coyote	-	-
MC2-146	M	Crawford	2/19/2017	A	Coyote	-	-
MC2-147	F	Crawford	2/19/2017	A	Coyote	-	+
MC2-150	F	Cambria	2/19/2017	A	Coyote	-	-
MC2-155	M	Centre	2/19/2017	A	Coyote	-	-

MC2-157	NR	NR	2/19/2017	NR	Coyote	-	+
MC2-170	F	Erie	2/19/2017	A	Coyote	+	+
MC2-174	NR	NR	2/19/2017	NR	Coyote	-	-
MC2-179	F	Warren	2/19/2017	A	Coyote	-	-
MC2-020	F	Clearfield	2/17/2017	J	Coyote	-	+
MC2-021	M	Crawford	2/18/2017	SA	Coyote	-	+
MC2-023	F	Centre	2/18/2017	A	Coyote	-	+
MC2-024	F	Tioga	2/18/2017	SA	Coyote	-	+
MC2-025	M	Warren	2/18/2017	A	Coyote	-	+
MC2-026	M	Warren	2/18/2017	A	Coyote	-	+
MC2-028	F	Clarion	2/18/2017	J	Coyote	-	-
MC2-029	F	Venango	2/18/2017	SA	Coyote	-	-
MC2-033	M	Centre	2/18/2017	J	Coyote	-	-
MC2-034	M	Centre	2/18/2017	SA	Coyote	+	-
MC2-035	M	Clearfield	2/18/2017	SA	Coyote	+	-
MC2-036	F	Bedford	2/19/2017	SA	Coyote	-	+
MC2-037	M	Bedford	2/19/2017	J	Coyote	-	-
MC2-039	M	Huntingdon	2/19/2017	SA	Coyote	-	+
MC2-041	M	Warren	2/19/2017	SA	Coyote	-	+
MC2-044	F	Monroe	2/19/2017	SA	Coyote	-	-
MC2-047	M	Elk	2/19/2017	SA	Coyote	-	-
MC2-048	NR	NR	2/19/2017	NR	Coyote	-	-
MC2-051	NR	NR	2/19/2017	NR	Coyote	-	-
MC2-052	M	Clarion	2/19/2017	A	Coyote	-	+
MC2-62	F	Somerset	2/19/2017	A	Coyote	-	-
MC2-65	M	Clearfield	2/19/2017	A	Coyote	-	+
MC2-68	M	Northumberland	2/19/2017	A	Coyote	-	-
MC2-70	F	Northumberland	2/19/2017	SA	Coyote	-	-
MC2-73	M	Pike	2/19/2017	SA	Coyote	-	+
MC2-76	F	Centre	2/19/2017	A	Coyote	-	+
MC2-79	F	Crawford	2/19/2017	J	Coyote	-	-

MC2-80	M	Armstrong	2/19/2017	J	Coyote	-	+
MC2-82	F	Allegheny	2/19/2017	A	Coyote	+	-
MC2-83	F	Clarion	2/19/2017	J	Coyote	+	-
MC2-89	M	Sullivan	2/19/2017	A	Coyote	-	+
MC2-92	F	Montour	2/19/2017	SA	Coyote	+	+
MC2-95	M	Bradford	2/19/2017	SA	Coyote	+	-
MC22	M	Cumberland	2/22/2015	SA	Coyote	-	-
MC26	M	Clearfield	2/22/2015	A	Coyote	+	+
MC28	F	Clearfield	2/22/2015	J	Coyote	+	+
MC30	M	Potter	2/22/2015	A	Coyote	+	+
MC32	F	Fayette	2/22/2015	J	Coyote	-	-
MC35	M	Beaver	2/22/2015	J	Coyote	-	-
MC40-1	M	Erie	2/22/2015	A	Coyote	+	-
MC40-2	M	Centre	2/22/2015	SA	Coyote	+	-
MC48	M	Tioga	2/22/2015	SA	Coyote	+	+
MC49	M	McKean	2/22/2015	A	Coyote	+	-
MC53	F	Clinton	2/22/2015	J	Coyote	-	+
MC56	M	Washington	2/22/2015	A	Coyote	+	+
MC57	F	Allegheny	2/22/2015	SA	Coyote	-	-
MC86	F	Northumberland	2/22/2015	A	Coyote	-	+
MC87	F	Pike	2/22/2015	A	Coyote	-	+
MC90	M	Wyoming	2/22/2015	A	Coyote	-	-
MC97	F	Mercer	2/22/2015	A	Coyote	-	+
MC99	F	Mercer	2/22/2015	J	Coyote	+	-
SP0	NR	NR	NR	NR	Coyote	-	-
SP02	M	Wyoming	1/23/2015	SA	Coyote	-	+
SP03	M	Luzerne	1/23/2015	A	Coyote	-	+
SP10	F	Susquehanna	1/24/2015	A	Coyote	-	-
SP11	M	Wayne	1/24/2015	A	Coyote	-	-
SP12	F	Wayne	1/24/2015	A	Coyote	-	-
SP14	F	Wayne	1/24/2015	A	Coyote	-	-

SP15	F	Wayne	1/24/2015	A	Coyote	-	+
SP17	M	Susquehanna	1/24/2015	SA	Coyote	-	+
SP18	M	Bradford	1/24/2015	A	Coyote	-	+
SP19	F	Bradford	1/24/2015	A	Coyote	-	-
SP20	M	Wyoming	1/25/2015	A	Coyote	-	+
SP21	M	Luzerne	1/25/2015	A	Coyote	-	+
SP24	F	Susquehanna	1/25/2015	A	Coyote	-	-
SP25	M	Susquehanna	1/25/2015	A	Coyote	-	+
SP26	F	Wyoming	1/25/2015	SA	Coyote	-	-
T1	F	Pike	1/24/2020	J	Coyote	+	+
T2	M	Pike	1/24/2020	SA	Coyote	+	-
T3	F	Susquehanna	1/24/2020	J	Coyote	-	-
T4	M	Wyoming	1/24/2020	A	Coyote	-	+
T6	M	Luzerne	1/24/2020	A	Coyote	-	-
T7	F	Luzerne	1/24/2020	A	Coyote	-	+
T8	F	Susquehanna	1/24/2020	A	Coyote	-	+
T9	F	Wyoming	1/24/2020	A	Coyote	-	+
T11	M	Wayne	1/24/2020	J	Coyote	-	-
T12	F	Wayne	1/24/2020	J	Coyote	-	-
T15	M	Bradford	1/24/2020	A	Coyote	+	-
T17	M	Bradford	1/24/2020	J	Coyote	-	+
T20	M	Bradford	1/24/2020	A	Coyote	-	+
T21	F	Luzerne	1/24/2020	A	Coyote	+	-
T23	F	Luzerne	1/24/2020	A	Coyote	-	+
T24	M	Lackawanna	1/24/2020	SA	Coyote	-	+
T26	M	Wayne	1/24/2020	SA	Coyote	+	+
T27	M	Wayne	1/24/2020	SA	Coyote	-	+
T28	F	Susquehanna	1/25/2020	SA	Coyote	-	-
T30	F	Susquehanna	1/25/2020	J	Coyote	-	+
T31	M	Wyoming	1/25/2020	SA	Coyote	-	+
T32	M	Lackawanna	1/25/2020	J	Coyote	+	+

T33	M	Wayne	1/25/2020	SA	Coyote	-	+
T34	F	Wayne	1/25/2020	J	Coyote	-	+
T35	M	Lackawanna	1/26/2020	J	Coyote	-	+
T39	M	Wayne	1/26/2020	A	Coyote	+	-
T40	F	Luzerne	1/26/2020	J	Coyote	-	-
T41	F	Wyoming	1/26/2020	A	Coyote	-	-
T42	F	Wyoming	1/26/2020	SA	Coyote	-	+
T44	M	Wayne	1/26/2020	SA	Coyote	-	+
T46	F	Lackawanna	1/25/2020	J	Coyote	-	+
S1	F	Susquehanna	1/17/2020	A	Coyote	-	+
S2	M	Susquehanna	1/17/2020	SA	Coyote	-	+
S3	M	Susquehanna	1/17/2020	SA	Coyote	-	-
S4	M	Susquehanna	1/17/2020	A	Coyote	-	+
S5	F	Luzerne	1/17/2020	A	Coyote	-	+
S6	M	Wayne	1/17/2020	J	Coyote	-	-
S7	F	Wayne	1/17/2020	J	Coyote	+	-
S8	M	Wayne	1/17/2020	J	Coyote	+	-
S9	F	Lackawanna	1/17/2020	J	Coyote	-	-
S13	F	Susquehanna	1/18/2020	J	Coyote	-	-
S17	M	Susquehanna	1/18/2020	A	Coyote	-	-
S18	F	Wayne	1/18/2020	J	Coyote	+	+
S19	M	Susquehanna	1/18/2020	SA	Coyote	-	+
S21	F	Bradford	1/18/2020	A	Coyote	+	+
S22	M	Bradford	1/18/2020	SA	Coyote	-	+
S24	M	Susquehanna	1/19/2020	A	Coyote	-	+
S25	F	Susquehanna	1/19/2020	J	Coyote	-	-
S26	M	Susquehanna	1/19/2020	A	Coyote	-	-
S27	M	Wyoming	1/19/2020	A	Coyote	-	+
S28	F	Wyoming	1/19/2020	A	Coyote	-	-
S29	M	Wyoming	1/19/2020	J	Coyote	-	-
S30	M	Wayne	1/19/2020	SA	Coyote	-	+

S32	M	Wayne	1/19/2020	A	Coyote	-	-
S34	M	Wayne	1/19/2020	SA	Coyote	-	-
S35	M	Wayne	1/19/2020	A	Coyote	+	+
C3	M	Armstrong	2/7/2020	SA	Coyote	+	-
C4	M	Cambria	2/7/2020	J	Coyote	-	-
C7	M	Armstrong	2/7/2020	SA	Coyote	-	-
C8	F	Armstrong	2/7/2020	SA	Coyote	-	+
C17	F	Bedford	2/8/2020	A	Coyote	-	+
C18	F	Indiana	2/8/2020	SA	Coyote	-	-
C19	M	Cambria	2/8/2020	A	Coyote	-	-
C20	M	Indiana	2/8/2020	SA	Coyote	-	-
C21	F	Bedford	2/8/2020	A	Coyote	-	-
C22	F	Clearfield	2/8/2020	A	Coyote	-	+
C23	F	Clearfield	2/8/2020	A	Coyote	-	-
C24	F	Clearfield	2/8/2020	A	Coyote	-	+
C25	M	Clearfield	2/8/2020	A	Coyote	-	+
C26	F	Venango	2/8/2020	SA	Coyote	-	+
C27	F	Venango	2/8/2020	SA	Coyote	-	-
C28	F	Clearfield	2/8/2020	A	Coyote	-	+
C29	M	Somerset	2/9/2020	SA	Coyote	+	_
C31	M	Somerset	2/9/2020	J	Coyote	+	+
C33	M	Cambria	2/9/2020	N/A	Coyote	+	-
C42	F	Cambria	2/9/2020	A	Coyote	-	-
C47	M	Somerset	2/9/2020	A	Coyote	-	+
C50	M	Clearfield	2/9/2020	A	Coyote	-	-
C52	M	Indiana	2/8/2020	J	Coyote	-	+
C58	F	Cambria	2/8/2020	SA	Coyote	+	-
C61	F	Butler	2/9/2020	A	Coyote	-	-
C66	M	Tioga	2/9/2020	A	Coyote	+	+
C67	M	Cambria	2/9/2020	A	Coyote	-	-
C70	M	Erie	2/8/2020	A	Coyote	-	-

C71	M	Erie	2/8/2020	A	Coyote	-	-
C72	M	Allegheny	2/8/2020	J	Coyote	-	-
C74	F	Erie	2/8/2020	J	Coyote	-	-
C75	M	Allegheny	2/8/2020	SA	Coyote	-	+
C76	M	Allegheny	2/8/2020	SA	Coyote	-	-
C77	F	Warren	2/8/2020	J	Coyote	-	-
C78	F	Bradford	2/8/2020	J	Coyote	-	+
C80	F	Bradford	2/8/2020	A	Coyote	-	+
M1	M	Tioga	2/21/2020	SA	Coyote	-	+
M2	M	Clinton	2/21/2020	A	Coyote	-	+
M3	M	Erie	2/21/2020	A	Coyote	-	-
M4	M	Centre	2/21/2020	SA	Coyote	-	+
M5	M	Clearfield	2/21/2020	J	Coyote	-	-
M6	M	Clearfield	2/21/2020	J	Coyote	-	+
M13	F	Cameron	2/22/2020	J	Coyote	+	+
M14	M	Centre	2/22/2020	SA	Coyote	+	+
M15	M	Centre	2/22/2020	J	Coyote	-	+
M16	M	Clearfield	2/21/2020	J	Coyote	-	-
M17	F	Clearfield	2/21/2020	J	Coyote	+	-
M22	M	Elk	2/22/2020	SA	Coyote	+	-
M23	M	Warren	2/21/2020	SA	Coyote	-	+
M25	F	Clarion	2/22/2020	SA	Coyote	-	-
M26	F	Centre	2/22/2020	J	Coyote	+	-
M27	M	Centre	2/22/2020	SA	Coyote	+	-
M29	M	Cambria	2/22/2020	SA	Coyote	-	+
M30	M	Clarion	2/22/2020	SA	Coyote	-	-
M31	M	Clarion	2/22/2020	SA	Coyote	+	-
M35	M	Beaver	2/21/2020	J	Coyote	-	-
M39	M	Fayette	2/22/2020	J	Coyote	-	+
M40	F	Westmoreland	2/22/2020	J	Coyote	-	+
M42	M	Centre	2/21/2020	SA	Coyote	-	+

M44	F	Tioga	2/23/2020	J	Coyote	-	-
M46	M	Potter	2/22/2020	SA	Coyote	-	-
M50	F	Clarion	2/22/2020	J	Coyote	+	-
M51	F	Clarion	2/22/2020	SA	Coyote	-	+
M52	F	Lawrence	2/22/2020	SA	Coyote	-	-
M54	M	Clearfield	2/22/2020	A	Coyote	+	-
M55	M	Cumberland	2/22/2020	A	Coyote	-	-
M59	M	Jefferson	2/22/2020	A	Coyote	+	+
M66	F	Armstrong	2/22/2020	J	Coyote	-	-
M68	M	Lawrence	2/22/2020	SA	Coyote	+	-
M69	F	Lawrence	2/22/2020	SA	Coyote	-	-
M71	M	Elk	2/23/2020	A	Coyote	-	+
M74	M	Centre	2/21/2020	SA	Coyote	-	-
M77	F	Centre	2/22/2020	J	Coyote	-	-
M78	M	Centre	2/22/2020	SA	Coyote	-	+
M79	M	Lycoming	2/22/2020	A	Coyote	-	+
M81	M	Juniata	2/22/2020	SA	Coyote	+	-
M85	F	McKean	2/22/2020	SA	Coyote	-	+
M90	M	Allegheny	2/22/2020	SA	Coyote	+	+
M91	F	Elk	2/22/2020	SA	Coyote	-	-
M95	M	Greene	2/22/2020	A	Coyote	+	-
M96	M	Greene	2/22/2020	A	Coyote	-	-
M100	M	Clearfield	2/22/2020	J	Coyote	-	-
M103	M	Susquehanna	2/22/2020	SA	Coyote	-	-
M106	F	Lycoming	2/22/2020	SA	Coyote	+	-
S10	F	Wyoming	1/18/2020	A	Red Fox	-	-
S11	M	Luzerne	1/18/2020	A	Gray Fox	-	-
S14	M	Wyoming	1/18/2020	A	Gray Fox	-	+
S15	F	Lackawanna	1/18/2020	A	Red Fox	-	-
S16	M	Wyoming	1/18/2020	SA	Red Fox	-	+
A1	F	Adams	1/16/2020	A	Red Fox	+	-

A3	M	Adams	1/16/2020	SA	Red Fox	-	-
A4	M	Adams	1/16/2020	A	Red Fox	-	+
A5	M	Adams	1/16/2020	A	Red Fox	-	+
A7	F	Adams	1/16/2020	J	Red Fox	-	-
A8	M	Adams	1/16/2020	A	Red Fox	-	+
A12	M	Adams	1/16/2020	A	Red Fox	-	-
A13	M	Adams	1/16/2020	A	Red Fox	-	-
A14	F	Adams	1/16/2020	J	Red Fox	-	+
A17	F	Adams	1/16/2020	J	Red Fox	-	-
A19	F	Adams	1/16/2020	J	Red Fox	-	+
A20	M	Adams	1/16/2020	A	Red Fox	-	-
A21	M	Adams	1/16/2020	SA	Red Fox	-	-
A23	M	Adams	1/16/2020	A	Red Fox	+	-
A24	F	Adams	1/16/2020	J	Red Fox	-	-
A25	M	Adams	1/16/2020	A	Red Fox	-	+
A27	F	Adams	1/16/2020	J	Red Fox	-	-
A30	M	Adams	1/16/2020	J	Red Fox	-	+
A31	M	Adams	1/16/2020	A	Red Fox	+	-
A36	F	Adams	1/16/2020	A	Gray Fox	-	+
A37	M	Adams	1/16/2020	SA	Red Fox	+	-
A40	M	Adams	1/17/2020	SA	Red Fox	-	+
A45	F	Adams	1/17/2020	SA	Red Fox	+	+
A46	M	Adams	1/17/2020	SA	Red Fox	-	+
A48	F	Adams	1/17/2020	A	Red Fox	-	-
A50	F	Adams	1/17/2020	A	Red Fox	-	+
A52	F	Adams	1/17/2020	A	Gray Fox	-	+
C2	F	Somerset	2/7/2020	A	Gray Fox	-	-
C5	M	Blair	2/7/2020	A	Gray Fox	-	+
C6	F	Blair	2/7/2020	SA	Red Fox	-	+
C9	M	Butler	2/7/2020	A	Gray Fox	-	+
C12	F	Blair	2/8/2020	A	Gray Fox	-	-

C13	M	Blair	2/8/2020	A	Gray Fox	-	+
C15	F	Blair	2/8/2020	A	Gray Fox	-	+
C16	M	Centre	2/8/2020	A	Gray Fox	-	-
C30	M	Cambria	2/8/2020	J	Red Fox	-	-
C32	M	Westmoreland	2/9/2020	SA	Red Fox	+	-
C35	M	Clinton	2/9/2020	A	Gray Fox	-	+
C36	M	York	2/9/2020	A	Red Fox	-	-
C37	M	York	2/9/2020	A	Red Fox	+	+
C38	M	York	2/9/2020	SA	Red Fox	+	-
C39	F	York	2/9/2020	SA	Red Fox	+	+
C40	F	York	2/9/2020	A	Red Fox	-	-
C43	M	Cambria	2/9/2020	A	Gray Fox	-	-
C44	F	Huntingdon	2/9/2020	A	Red Fox	+	+
C45	M	Huntingdon	2/9/2020	SA	Red Fox	+	-
C46	F	Huntingdon	2/9/2020	J	Red Fox	-	+
C48	M	Indiana	2/9/2020	A	Gray Fox	-	+
C53	M	York	2/9/2020	SA	Red Fox	-	-
C56	M	York	2/9/2020	J	Red Fox	+	-
C59	F	Lancaster	2/9/2020	A	Red Fox	-	-
C60	M	York	2/9/2020	A	Red Fox	-	-
C63	F	Lancaster	2/9/2020	A	Red Fox	-	+
C64	M	Lancaster	2/9/2020	A	Red Fox	-	-
GF.CR.6	M	Clinton	2/9/2020	NR	Gray Fox	+	+
RF.CR.11	M	York	2/9/2020	NR	Red fox	-	+
RF.CR.12	F	York	2/9/2020	NR	Red Fox	-	+
RF.CR.13	F	York	2/9/2020	NR	Red Fox	+	+
RF.CR.14	M	York	2/9/2020	NR	Red Fox	+	-
GF.CR.25	NR	Cambria	2/9/2020	NR	Gray Fox	+	+
RF.CR.26	NR	Lancaster	2/9/2020	NR	Red Fox	+	+
RF.CR.34	M	York	2/9/2020	NR	Red Fox	+	+
RF.CR.35	M	Westmoreland	2/9/2020	NR	Red Fox	-	+

RF.CR.37	F	Cambria	2/9/2020	NR	Red Fox	+	-
RF.CR.39	M	York	2/9/2020	NR	Red Fox	-	+
RF.CR.41	M	York	2/9/2020	NR	Red Fox	+	-
RF.CR.42	M	Lancaster	2/9/2020	NR	Red Fox	+	+
RF.CR.43	M	Lancaster	2/9/2020	NR	Red Fox	+	+
RF.CR.44	F	Lancaster	2/9/2020	NR	Red Fox	+	+
RF.11.6.19	NR	Northumberland	11/6/2019	NR	Red Fox	-	+
RF.12.7.19	NR	Union	12/8/2019	NR	Red Fox	-	+
RF.1.5.20	NR	Lycoming	1/7/2020	NR	Red Fox	-	+
RF.1.6.20.A	NR	Juniata	1/7/2020	NR	Red Fox	+	+
RF.2.4.20	M	Bucks	2/4/2020	NR	Red Fox	+	+

Pennsylvania (PA); Male (M); Female (F); Not Reported (NR); Adult (A); Subadult (SA); Juvenile (J); Canine Distemper Virus (CDV); Canine Parvovirus (CPV); Antibody (Ab); Identifier (ID)

Supplementary Figure S1. Images of TiterCHEK CDV-CPV results

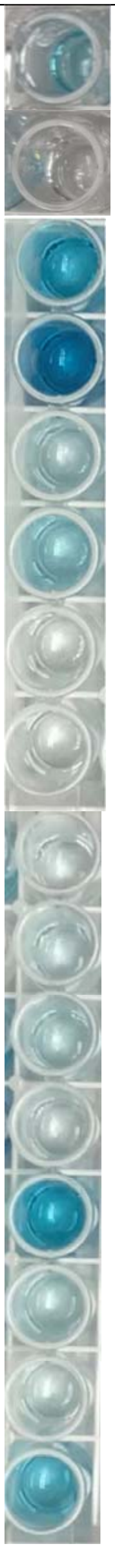
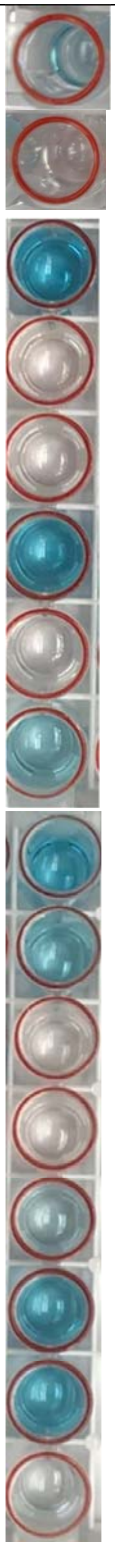

Coyote Serum ID	CDV	CPV
+	+	+
-	-	-
T1	+	+
T2	+	-
T3	-	-
T4	-	+
T6	-	-
T7	-	+
T8	-	+
T9	-	+
T11	-	-
T12	-	-
T15	+	-
T17	-	+
T20	-	+
T21	+	-

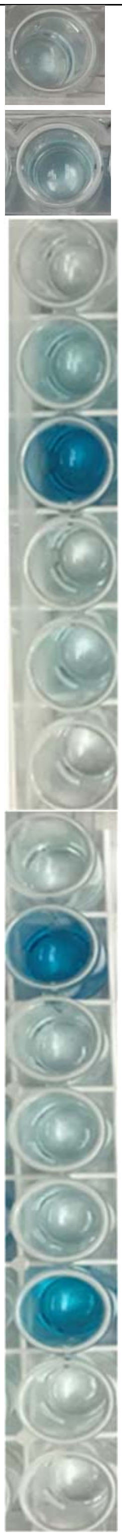
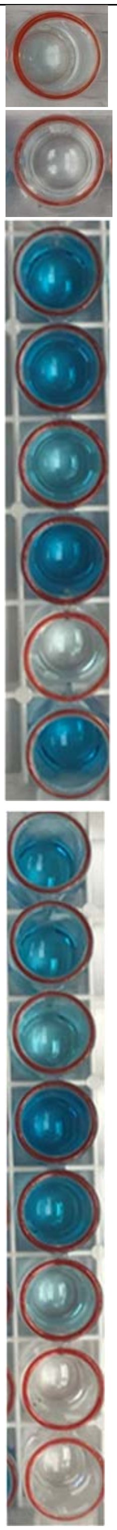

Coyote Serum ID	CDV	CPV
MC2-18	-	-
MC2-19	-	-
T23	-	+
T24	-	+
	+	+
T26	-	+
T27	-	-
T28	-	+
T30	-	+
T31	-	+
T32	+	+
T33	-	+
T34	-	+
T35	-	+
T39	+	-
T40	-	-
T41	-	-

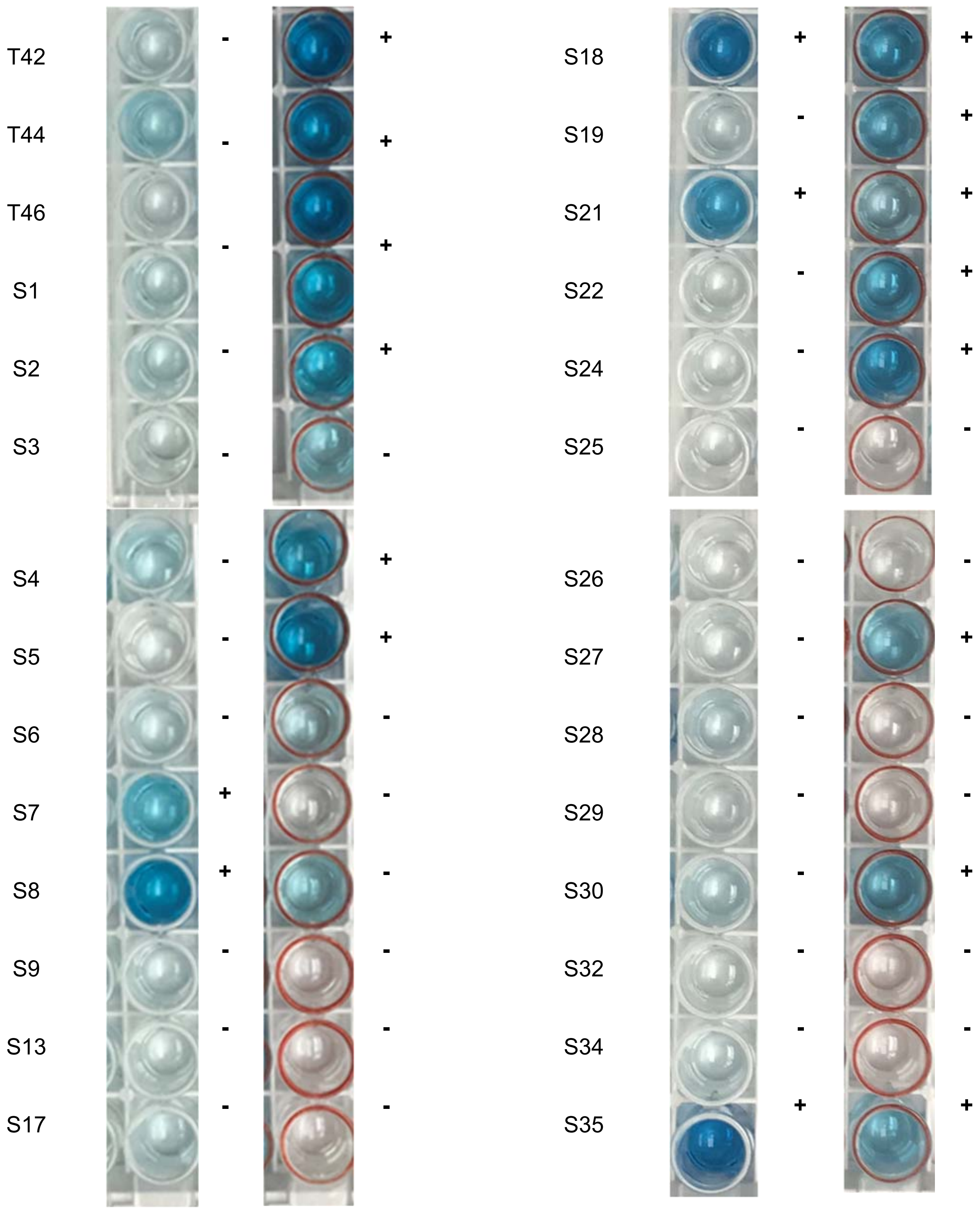
T42
T44
T46
S1
S2
S3
- +
- +
- +
- +
- -
S18 + +
S19 - +
S21 + +
S22 - +
S24 - +
S25 - -
S4 - +
S5 - +
S6 - -
S7 + -
S8 + -
S9 - -
S13 - -
S17 - -
S26 - -
S27 - +
S28 - -
S29 - -
S30 - +
S32 - -
S34 - -
S35 + +

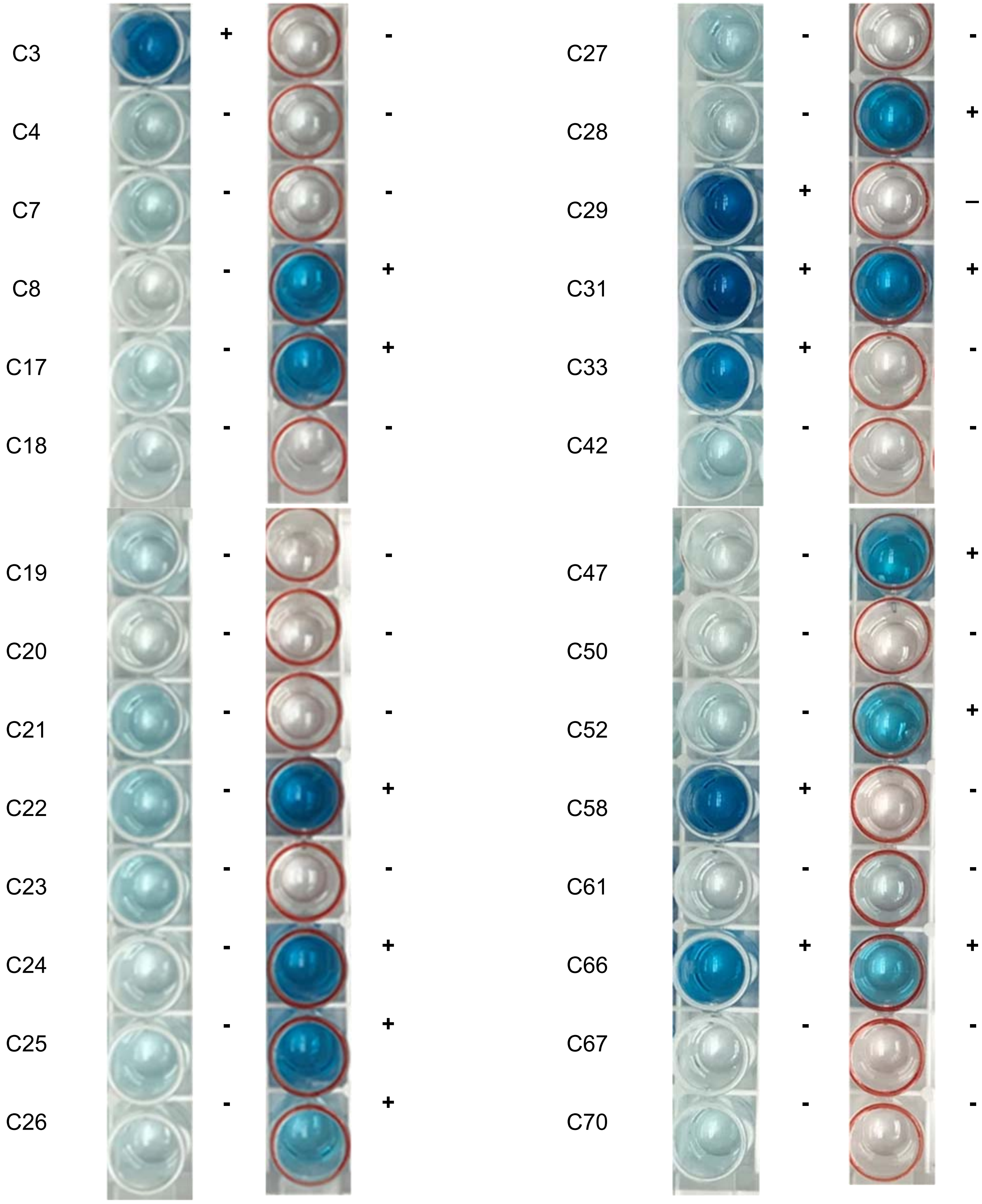

C3 + -
C4 - -
C7 - -
C8 - +
C17 - +
C18 - -
C19 - -
C20 - -
C21 - -
C22 - +
C23 - -
C24 - +
C25 - +
C26 - +
C27 - -
C28 - +
C29 + –
C31 + +
C33 + -
C42 - -
C47 - +
C50 - -
C52 - +
C58 + -
C61 - -
C66 + +
C67 - -
C70 - -

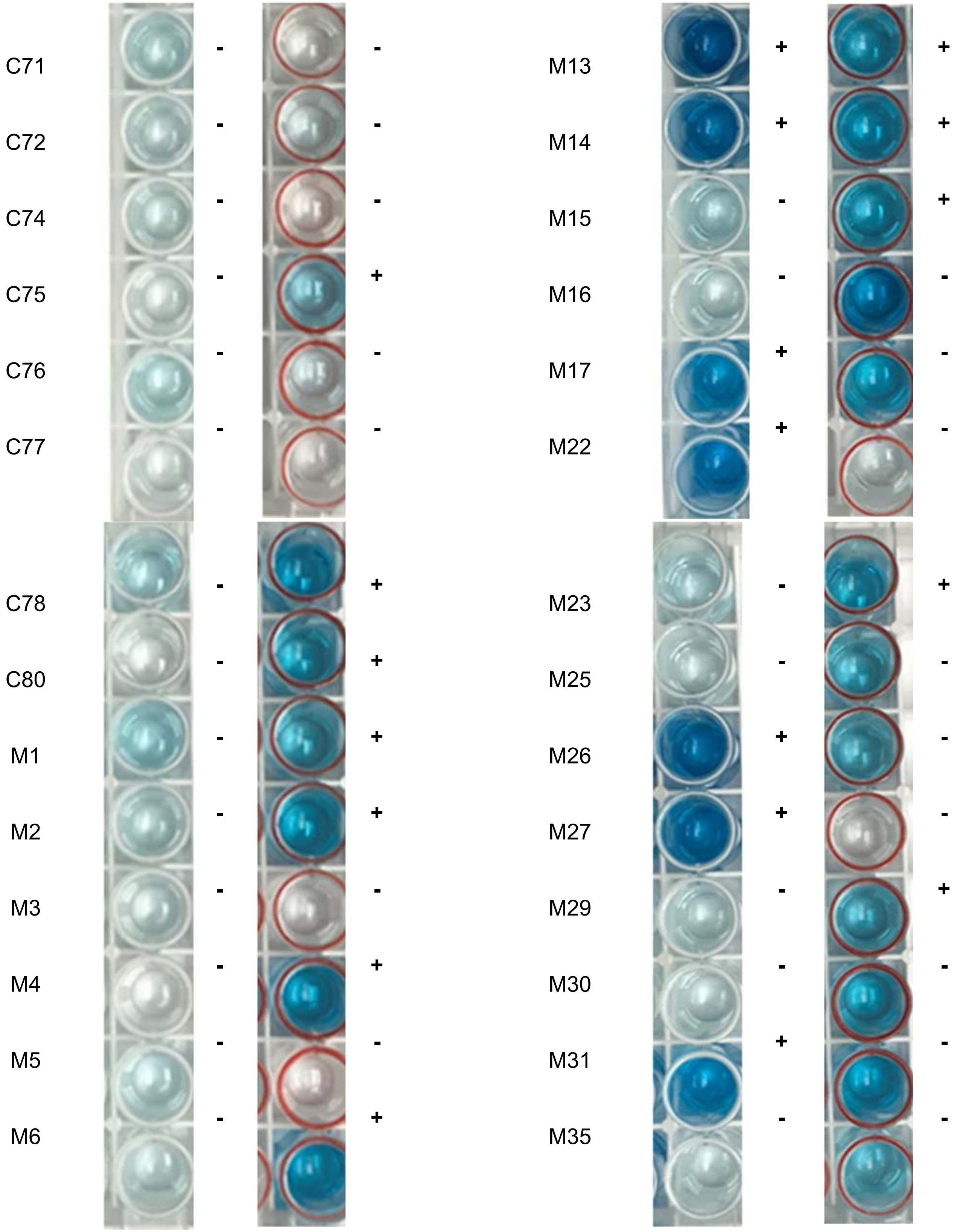

C71 - -
C72 - -
C74 - -
C75 - +
C76 - -
C77 - -
C78 - +
C80 - +
M1 - +
M2 - +
M3 - -
M4 - +
M5 - -
M6 - +
M13 + +
M14 + +
M15 - +
M16 - -
M17 + -
M22 + -
M23 - +
M25 - -
M26 + -
M27 + -
M29 - +
M30 - -
M31 + -
M35 - -

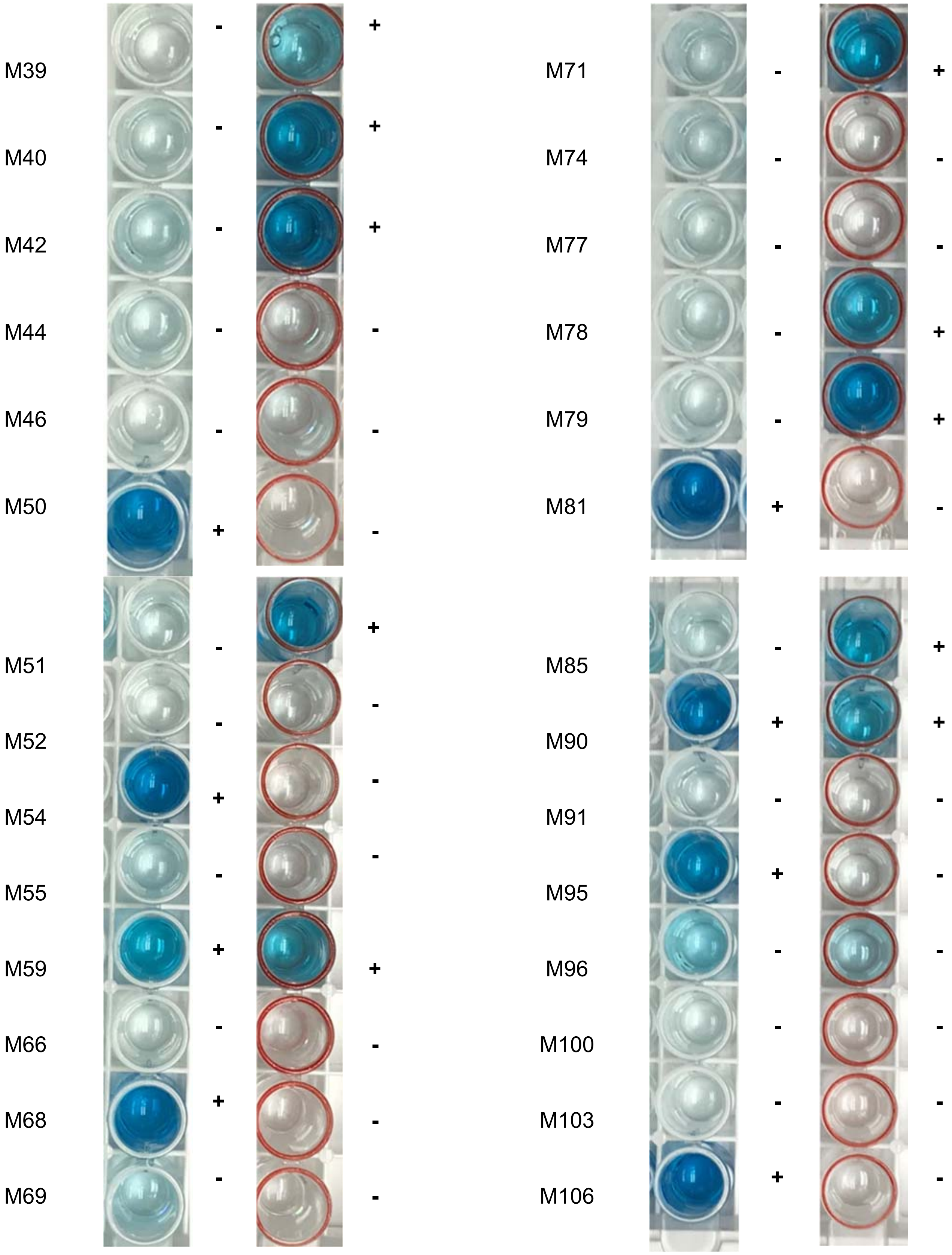

M39 - +
M40 - +
M42 - +
M44 - -
M46 - -
M50 + -
M51 - +
M52 - -
M54 + -
M55 - -
M59 + +
M66 - -
M68 + -
M69 - -
M71 - +
M74 - -
M77 - -
M78 - +
M79 - +
M81 + -
M85 - +
M90 + +
M91 - -
M95 + -
M96 - -
M100 - -
M103 - -
M106 + -

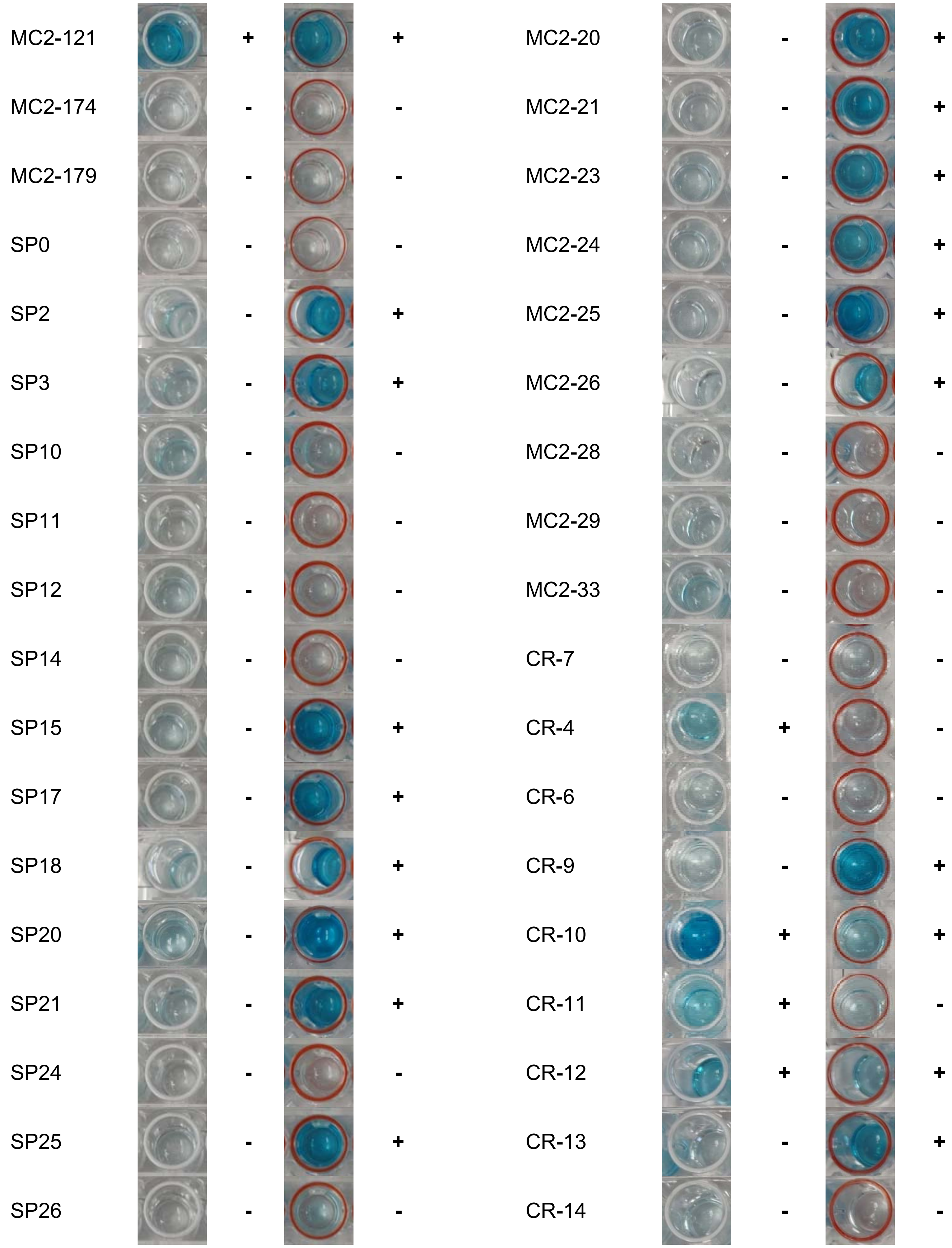
MC2-121 + +
MC2-174 - -
MC2-179 - -
SP0 - -
SP2 - +
SP3 - +
SP10 - -
SP11 - -
SP12 - -
SP14 - -
SP15 - +
SP17 - +
SP18 - +
SP20 - +
SP21 - +
SP24 - -
SP25 - +
SP26 - -
MC2-20 - +
MC2-21 - +
MC2-23 - +
MC2-24 - +
MC2-25 - +
MC2-26 - +
MC2-28 - -
MC2-29 - -
MC2-33 - -
CR-7 - -
CR-4 + -
CR-6 - -
CR-9 - +
CR-10 + +
CR-11 + -
CR-12 + +
CR-13 - +
CR-14 - -

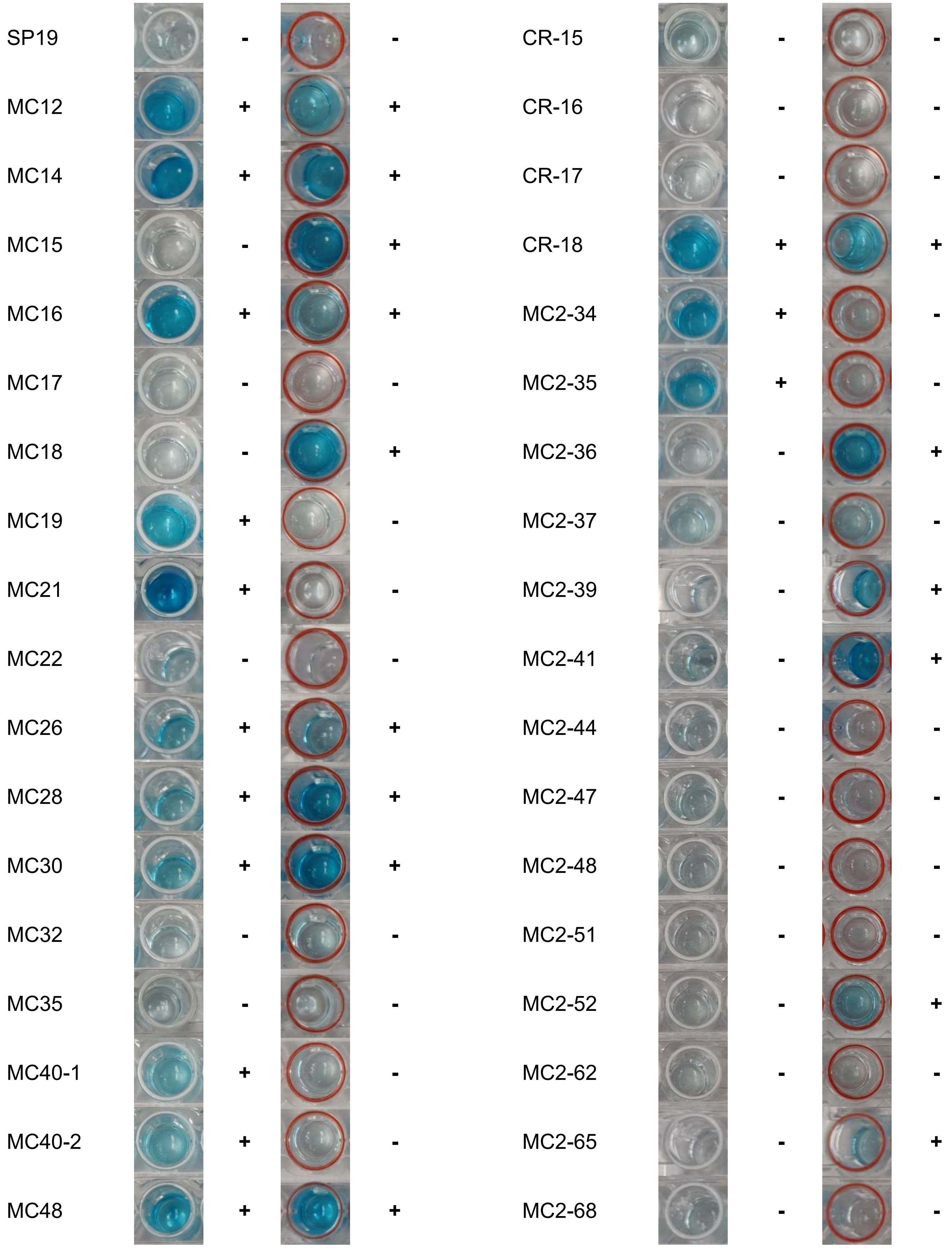
SP19 - -
MC12 + +
MC14 + +
MC15 - +
MC16 + +
MC17 - -
MC18 - +
MC19 + -
MC21 + -
MC22 - -
MC26 + +
MC28 + +
MC30 + +
MC32 - -
MC35 - -
MC40-1 + -
MC40-2 + -
MC48 + +
CR-15 - -
CR-16 - -
CR-17 - -
CR-18 + +
MC2-34 + -
MC2-35 + -
MC2-36 - +
MC2-37 - -
MC2-39 - +
MC2-41 - +
MC2-44 - -
MC2-47 - -
MC2-48 - -
MC2-51 - -
MC2-52 - +
MC2-62 - -
MC2-65 - +
MC2-68 - -

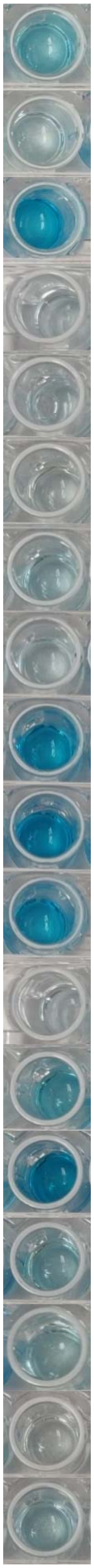
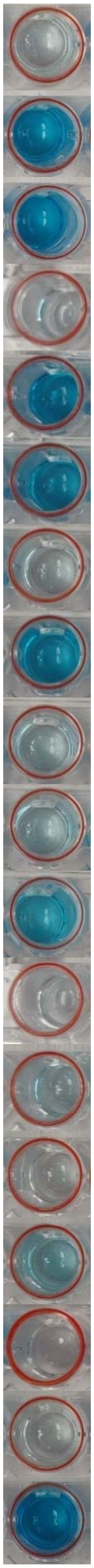
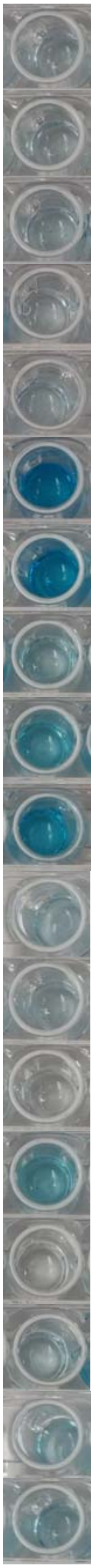
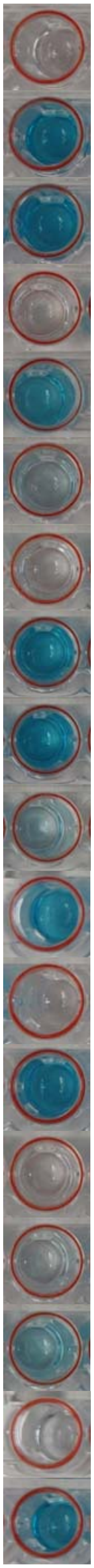

MC49	+	-	MC2-70	-	-
MC53	-	+	MC2-73	-	+
MC56	+	+	MC2-76	-	+
MC57	-	-	MC2-79	-	-
MC86	-	+	MC2-80	-	+
MC87	-	+	MC2-82	+	-
MC90	-	-	MC2-83	+	-
MC97	-	+	MC2-89	-	+
MC99	+	-	MC2-92	+	+
MC100	+	-	MC2-95	+	-
MC113	+	+	MC2-100	-	+
MC114	-	-	MC2-101	-	-
MC115	+	-	MC2-104	-	+
MC130	+	-	MC2-111	+	-
MC131	+	+	MC2-113	-	-
MC148	-	-	MC2-119	-	+
MC2-1	-	-	MC2-124	-	-
MC2-2	-	+	MC2-125	-	+

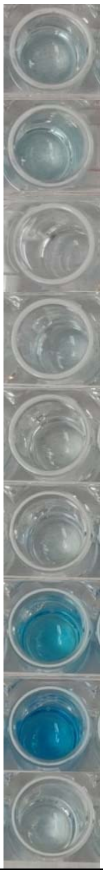
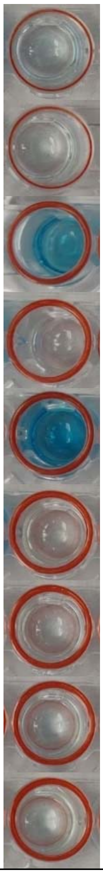

MC2-3	-	-
MC2-4	-	-
MC2-5	-	+
MC2-6	-	-
MC2-8	-	+
MC2-10	-	-
MC2-11	+	-
MC2-13	+	-
MC2-16	-	-

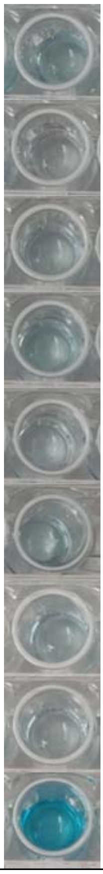
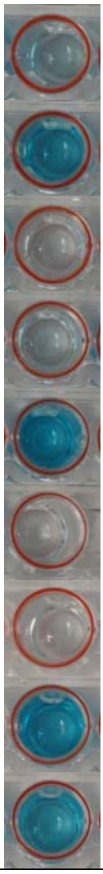

MC2-131	-	-
MC2-134	-	+
MC2-141	-	-
MC2-146	-	-
MC2-147	-	+
MC2-150	-	-
MC2-155	-	-
MC2-157	-	+
MC2-170	+	+

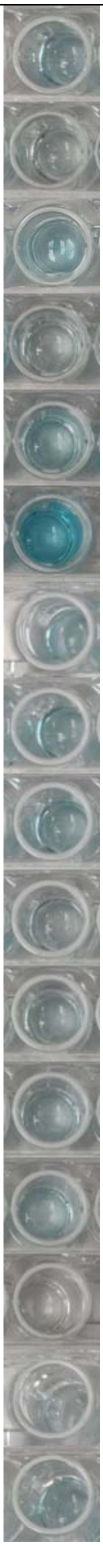
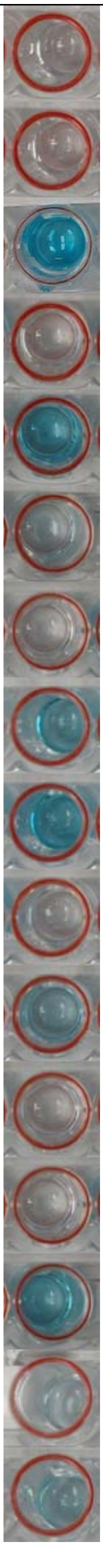
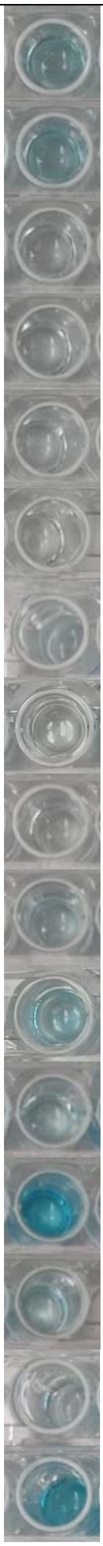
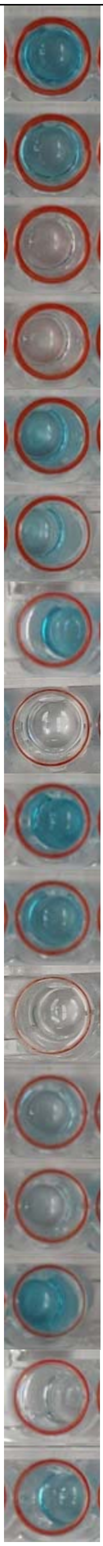

Fox Serum ID	CDV	CPV
S10	-	-
S11	-	-
S14	+	+
S15	-	-
S16	-	+
A1	+	-
A3	-	-
A4	-	+
A5	-	+
A7	-	-
A8	-	+
A12	-	-
A13	-	-
A14	-	+
A17	-	-
A19	-	+
A50	-	+
A52	-	+
52B	-	-
C2	-	-
C5	-	+
C6	-	+
C9	-	+
C12	-	-
C13	-	+
C15	-	+
C16	-	-
C30	-	-
C32	+	-
C35	-	+
C36	-	-
C37	+	+

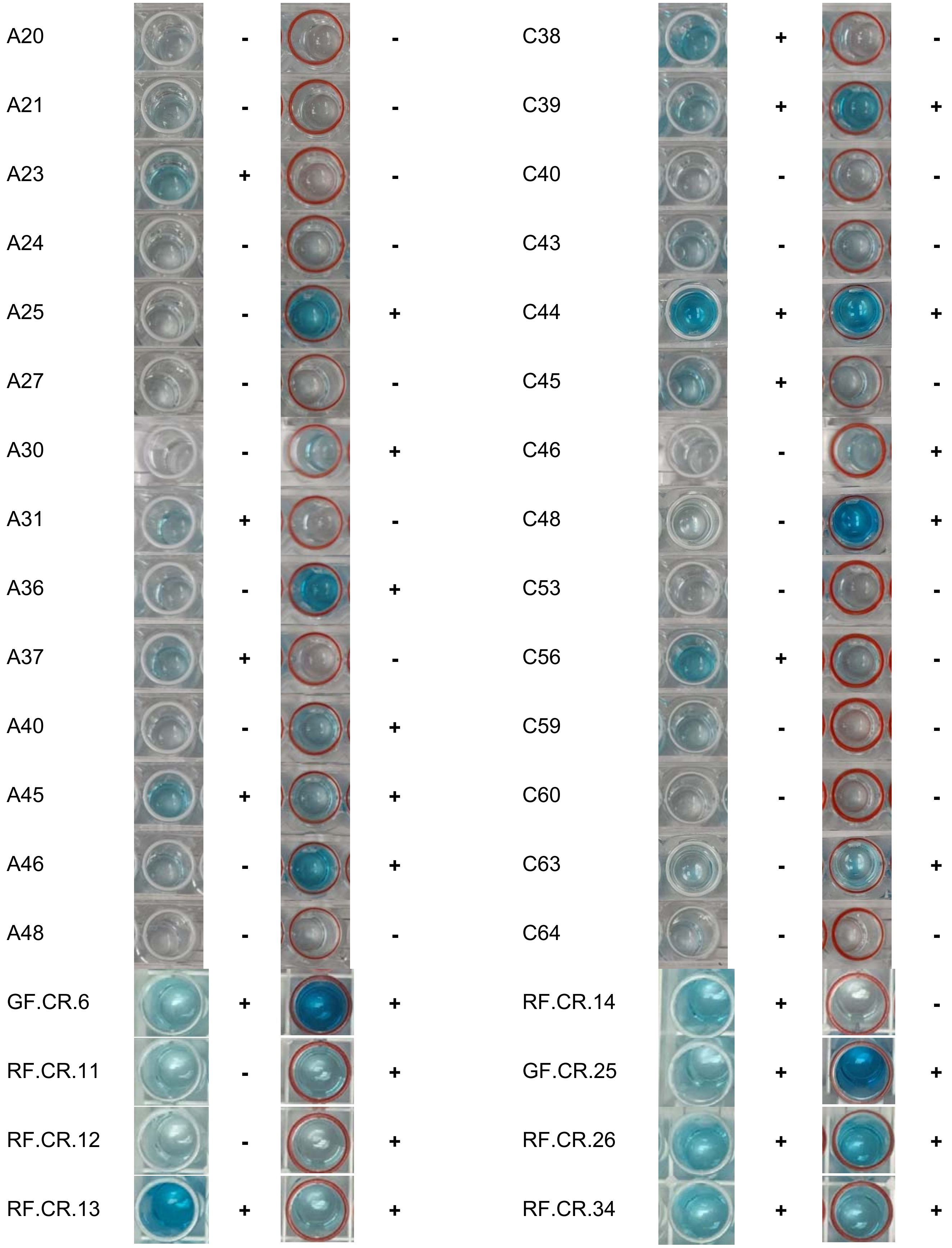
A20 - -
A21 - -
A23 + -
A24 - -
A25 - +
A27 - -
A30 - +
A31 + -
A36 - +
A37 + -
A40 - +
A45 + +
A46 - +
A48 - -
GF.CR.6 + +
RF.CR.11 - +
RF.CR.12 - +
RF.CR.13 + +
C38 + -
C39 + +
C40 - -
C43 - -
C44 + +
C45 + -
C46 - +
C48 - +
C53 - -
C56 + -
C59 - -
C60 - -
C63 - +
C64 - -
RF.CR.14 + -
GF.CR.25 + +
RF.CR.26 + +
RF.CR.34 + +

RF.CR.35	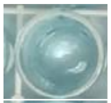	-	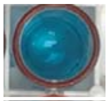	+
RF.CR.37	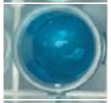	+	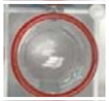	-
RF.CR.39	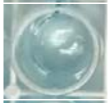	-	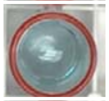	+
RF.CR.41	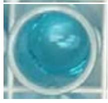	+	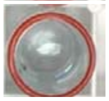	-
RF.CR.42	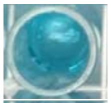	+	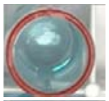	+
RF.CR.43	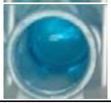	+	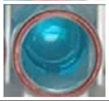	+

RF.CR.44	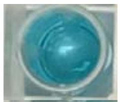	+	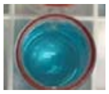	+
RF.11.6.19	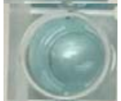	-	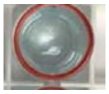	+
RF.12.7.19	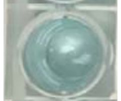	-	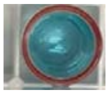	+
RF.1.5.20	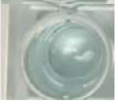	-	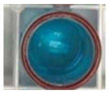	+
RF.1.6.20	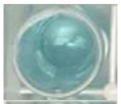	+	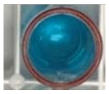	+
RF.2.4.20	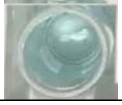	+	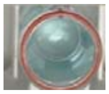	+
